# Lupin protein positively affects plasma LDL cholesterol and LDL:HDL cholesterol ratio in hypercholesterolemic adults after four weeks of supplementation: a randomized, controlled crossover study

**DOI:** 10.1186/1475-2891-12-107

**Published:** 2013-08-01

**Authors:** Melanie Bähr, Anita Fechner, Julia Krämer, Michael Kiehntopf, Gerhard Jahreis

**Affiliations:** 1 Institute of Nutrition, Department of Nutritional Physiology, Friedrich Schiller University Jena, Dornburger Str. 24, D-07743, Jena, Germany; 2 Institute of Clinical Chemistry and Laboratory Medicine, Jena University Hospital, Erlanger Allee 101, D-07747, Jena, Germany

**Keywords:** Human study, Lupin protein, Plasma lipids, Hypercholesterolemic subjects, Serum amino acids

## Abstract

**Background:**

A couple of studies indicate a favorable impact of lupin protein on cardiovascular risk factors in humans. These studies, however, used relatively high doses of > 33 g/d, which can hardly be consumed under physiological conditions. Therefore, we investigated the effect of 25 g/d lupin protein isolate (LPI) on selected cardiovascular markers and on serum amino acids.

**Methods:**

A total of 33 hypercholesterolemic subjects participated in a randomized, controlled, double-blind crossover study. LPI and the active comparator milk protein isolate (MPI) were incorporated in protein drinks and consumed over 8 wk separated by a 4 wk washout period. Anthropometric data, blood pressure, and nutrient intake were assessed at baseline and after 8 wk of both protein interventions. Blood was sampled at baseline, wk 4 and wk 8. All 33 subjects were included in final statistical analyses using repeated measures ANOVA with the general linear model or using linear mixed model.

**Results:**

Except for higher HDL cholesterol at wk 4 of LPI (*P* ≤ 0.036), anthropometric parameters, blood pressure, and plasma lipids did not differ among LPI and MPI intervention. Compared to baseline, the primary outcome LDL cholesterol was significantly reduced after 4 wk of both interventions (*P* ≤ 0.008), while LDL:HDL cholesterol ratio was decreased only by LPI (*P* = 0.003). These time effects were restricted to subjects with higher hypercholesterolemia and disappeared after 8 wk. Blood pressure was reduced after 8 wk of LPI (*P* ≤ 0.044). Almost all serum amino acids were higher at wk 4 but not at wk 8 of MPI compared to LPI. Following 4 wk and 8 wk of LPI intervention, most amino acids remained unchanged. Both interventions caused a slight, but significant rise in body weight and body fat after 8 wk (*P* ≤ 0.045).

**Conclusion:**

In conclusion, 25 g LPI can beneficially modulate plasma LDL cholesterol at least over short-term. Using appropriate dietetic conditions that improve consumer compliance and avoid changes in energy intake as well as in body composition, lupin protein could positively impact cardiovascular risk factors particularly in individuals with higher hypercholesterolemia.

**Trial registration:**

ClinicalTrials.gov: NCT01304992

## Background

Replacing of animal by plant protein in foods is currently an important topic of discussion due to the ecological and physiological benefits associated with vegetable sources of proteins. In view of the growing global population as well as the limited availability of agricultural land, there is an urgent need for high quality proteins from sustainable plant sources such as legumes (*e*.*g*., soy, pea, and lupin). Moreover, the rising incidence of cardiovascular diseases increases the demand for potential dietary interventions that could reduce the related risk factors. For established cardiovascular conditions such as coronary heart diseases and also for subjects at high risk, drug therapy is the recommended form for reducing elevated LDL cholesterol concentrations [1]. However, ancillary to an existing therapy or for the primary prevention of coronary heart diseases, non-pharmacological strategies such as weight reduction, increased physical activity, and healthier dietary habits are endorsed by the National Cholesterol Education Program (NCEP) [1].

Dietary proteins from plant sources can exert nutraceutical activities such as reduce blood lipids and lower blood pressure [2-5], and thus constitute a healthier diet. As reviewed by Sirtori *et al*. [2], investigations in animals have revealed that proteins derived from either white lupin (*Lupinus albus*) or blue lupin (*Lupinus angustifolius*) improve the lipoprotein profile and lower blood pressure. Most of the studies in humans evaluated the physiological effects of lupin flour or lupin fiber, and only a small number of investigations focused on the effects of lupin protein [6]. These studies observed a beneficial influence of lupin protein on blood cholesterol concentrations [7-9] and also partially on blood pressure [7]. Furthermore, the study by Naruszewicz *et al*. [7] revealed a significant reduction of the inflammatory marker “high-sensitivity C-reactive protein” (hs-CRP) after 90 d of lupin protein intake in hypercholesterolemic subjects. As shown in a prospective study in women, hs-CRP is a strong predictor of the risk of cardiovascular events [10]. The effect of lupin protein on the distribution of serum amino acids has only been examined in one human trial [8]. Most importantly, in all of these studies, relatively high doses comprising more than 30 g/d lupin protein were administered [7-9]. Such high daily doses can hardly be consumed under normal physiological conditions. For example, for soy protein, which is closely related to lupin protein, the US Food and Drug Administration established a health claim in 1999 stating that the intake of 25 g/d soy protein beneficially affects serum lipids in humans [11]. Thus, further studies are needed to firstly, evaluate the impact of an equivalent modest amount of lupin protein on cardiovascular health and secondly, to clarify the effect on serum amino acids.

Therefore, we conducted a randomized crossover intervention study to determine the impact of 25 g/d supplemental lupin protein isolate (LPI) compared to milk protein isolate (MPI) incorporated into protein drinks on cardiovascular markers (blood lipids, hs-CRP, and blood pressure) and on the amino acid profile in hypercholesterolemic subjects over an 8 wk period.

## Methods

### Subjects

A total of 65 volunteers aged between 18 and 80 years were recruited in the region of Jena. Eligibility criterion was a total cholesterol concentration of ≥ 5.2 mmol/L at screening, determined either by a general practitioner or on-site using a hand-held point of care device from capillary blood (Accutrend® Plus System, Roche Diagnostics, Grenzach-Wyhlen, Germany). Exclusion criteria were treatment with lipid-lowering drugs, intake of nutritional supplements, which potentially influence lipid metabolism, and intolerance, allergy or a strong dislike to any food ingredient present in the protein drinks used in the study. In addition, breast-feeding mothers or pregnant females were excluded. Thus, 33 eligible participants (18 females, 15 males) were invited to an in-person meeting. Here, participants were offered essential study-relevant information and also provided with a study folder containing print information. Written informed consent was obtained from all subjects before start of the study. The study protocol was approved by the Ethics Committee of the Medical Faculty of the Friedrich Schiller University, Jena (no.: 2607-07/09).

### Study design

**Figure 1:**
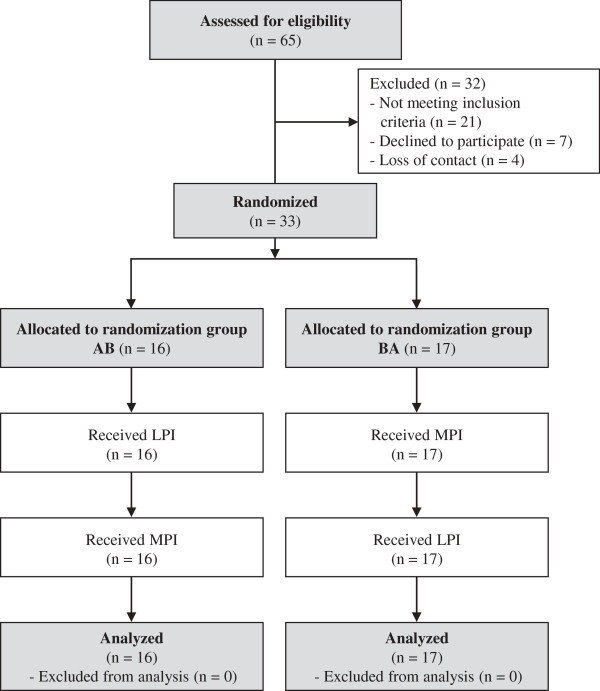
**Flow chart of participants at each stage of the intervention study.** LPI, lupin protein isolate; MPI, milk protein isolate.

The current study was part of a larger investigation consisting of two intervention studies examining the influence of two different daily doses of LPI: 25 g (present study) and 40 g [12] and comparing these with the effects of the respective doses of MPI. The present study used a randomized, double-blind crossover design consisting of two 8 wk intervention periods separated by a 4 wk washout period. The study was conducted between March and August 2011 at the Department of Nutritional Physiology, Friedrich Schiller University of Jena. Before commencement subjects were randomly assigned to one of two randomization groups using computer-generated random numbers. One group received LPI to start with (group AB) and the other group MPI first (group BA, Figure 1). Research assistants involved in the randomization procedure did not have access to any information regarding demographic or laboratory characteristics of the subjects. Moreover, protein drinks were labeled with numeric codes and all research assistants as well as the participants were blinded to group assignments.

### Study products

The daily portion of 25 g LPI or MPI was dissolved in 500 mL water. The protein drinks produced by Nutrichem diät + pharma GmbH (Roth, Germany) were assembled as a 4 wk supply in 250 mL sealed packages. Thus, 100 mL of the protein drink contained either 5.0 g LPI or the *iso*-nitrogenous amount of 5.1 g MPI. The amino acid compositions of the protein drinks are shown in Table 1. Fat (3.0 g/100 mL) and carbohydrates (3.5 g/100 mL) were added to obtain a pleasant taste and texture and to mask potential differences between the protein drinks. Subjects were instructed to maintain their usual level of physical activity as well as their dietary habits throughout the whole study. However, subjects were advised to replace an *iso*-caloric part of their usual diet with the protein drinks since these provided an additional energy intake of 1190 kJ/d.

**Table 1:** Relative amino acid composition of the two protein drinks administered in the study

**Amino acid (%)**	**LPI drink**	**MPI drink**
	**n = 3**	**n = 3**
Alanine	3.2 ± 0.2	3.0 ± 0.1
Arginine	10.9 ± 0.5	3.3 ± 0.1
Aspartate + asparagine	10.4 ± 0.5	7.1 ± 0.2
Cystine	1.4 ± 0.3	0.8 ± 0.1
Glutamate + glutamine	23.2 ± 1.1	21.2 ± 0.3
Glycine	4.0 ± 0.2	1.8 ± 0.1
Histidine	2.4 ± 0.1	2.5 ± 0.1
Isoleucine	3.8 ± 0.3	4.4 ± 0.1
Leucine	7.3 ± 0.3	9.2 ± 0.2
Lysine	4.2 ± 0.2	8.0 ± 0.1
Methionine	0.4 ± 0.1	2.5 ± 0.2
Phenylalanine	3.9 ± 0.2	4.6 ± 0.2
Proline	8.8 ± 3.8	9.4 ± 1.4
Serine	5.0 ± 0.3	5.5 ± 0.1
Threonine	3.2 ± 0.3	4.8 ± 0.5
Tryptophan	1.2 ± 1.2	1.5 ± 0.6
Tyrosine	3.6 ± 0.4	5.0 ± 0.2
Valine	3.3 ± 0.2	5.6 ± 0.1

Values are presented as mean ± standard deviation.

Abbreviation: *LPI* lupin protein isolate, *MPI* milk protein isolate.

The LPI was provided by the Fraunhofer Institute for Process Engineering and Packaging (Fh-IVV, Freising, Germany) in the form of the protein isolate type E. It was produced from the seeds of *Lupinus angustifolius cv*. Boregine as described by D'Agostina *et al*. [13]. This LPI contained 81.6 ± 1.3% protein (nitrogen × 5.8) and low quantities of water (5.7 ± 0.0%), ash (4.4 ± 0.0%), fiber (6.0 ± 0.4%), and fat (1.4 ± 0.1%) in fresh matter. In general, LPI type E contains the conglutins α, β, and δ [14] and is almost free of conglutin γ. Furthermore, it has low quantities of alkaloids represented only by lupanine (34.1 ± 3.1 mg/kg) [15].

In order to compare LPI with a high quality protein of similar palatability, a MPI consisting of a mixture of 75%:25% (wt%:wt%) sodium caseinate (EM7, DMV International, Veghel, The Netherlands) and whey protein Megglosat HP (ME, Meggle, Wasserburg, Germany) was chosen as active comparator. Sodium caseinate was made up of 88.9 ± 1.0% protein (nitrogen × 6.38), water (5.9 ± 0.0%), ash (4.4 ± 0.0%), fiber (2.8 ± 0.1%), and fat (0.6 ± 0.0%) in fresh matter. Megglosat HP consisted of 84.8 ± 2.6% protein (nitrogen × 6.38), water (6.6 ± 0.1%), ash (4.9 ± 0.0%), fiber (3.6 ± 0.0%), and fat (1.3 ± 0.2%) in fresh matter. The nutrient composition of the protein isolates was analyzed by applying standard methods with reference to the “Association of Analytical Communities” [16] and the European Community Directive [17].

### Data collection

At baseline participants recorded their usual daily eating patterns in a 5 d food record with a precise documentation of weights and types of all consumed foods and beverages. The composition of this basal diet was estimated with the use of PRODI® 5.9 (Nutri-Science GmbH, Freiburg, Germany). At the end of each intervention period, subjects consumed a standard diet including the protein drinks. This standard diet was prepared and preweighed in the study center and contained all foods required per subject over 2 d. Subjects were instructed to consume no other foods, except for water. Food intake was calculated by weighing food residues.

Body weight, body composition as well as blood pressure were determined at baseline and after 8 wk of each intervention period. Fasting participants were weighed with light clothes and without shoes using a digital scale. Body composition was determined using bioelectrical impedance analysis (BIA 2000-S, Data Input GmbH, Darmstadt, Germany). Blood pressure was measured in a sitting position in duplicate after 10 min of rest on the left arm using an automatic blood pressure monitor (boso-medicus uno, Bosch + Sohn GmbH u. Co. KG, Jungingen, Germany).

Blood samples were collected at baseline, and after 4 wk and 8 wk of each intervention period. Following 12 h overnight fasting, blood samples were drawn by venipuncture into a serum gel tube and a plasma gel tube containing lithium heparin (Sarstedt AG & Co., Nümbrecht, Germany). Serum tubes were centrifuged at 20°C, 2500 × *g* for 10 min, the serum supernatants were aliquoted and stored at –80°C until analysis. Plasma gel tubes were centrifuged at 15°C, 4302 × *g* for 7 min.

### Analytical methods

Fresh plasma was analyzed for total, LDL, and HDL cholesterol as well as for triacylglyceroles, urea, and hs-CRP according to the protocols of the Institute of Clinical Chemistry and Laboratory Medicine, Jena University Hospital and quantified using the autoanalyzer ARCHITECT C16000 (Abbott, Illinois, USA). For the analysis of free amino acids in serum, the method based on the European Community Directive [17] was applied as described previously [18].

### Statistical analyses

Statistical analyses were conducted using PASS 6.0 (NCSS Statistical Software, Kaysville, UT, USA) or SPSS 19.0 (SPSS Inc., Chicago, USA). In all analyses, differences were considered as statistically significant with *P* ≤ 0.050. A power analysis revealed > 80% power for the present study to detect a 10% difference in the primary outcome measure LDL cholesterol. All collected data were tested for normal distribution and for homogeneity of variances applying the Kolmogorov-Smirnov test and the Levene’s test, respectively. Baseline characteristics and data of the nutrient intake were tested with the independent samples *t*-tests. A repeated measures ANOVA with the general linear model was used to identify differences between the two treatments as well as changes over time. For data that were not normally distributed and/or had heterogeneous variances, a linear mixed model analysis was applied.

## Results

### Baseline characteristics and palatability of study products

All 33 individuals randomized in groups AB and BA completed both 8 wk intervention periods and were included in final analyses (Figure 1). The baseline characteristics of the subjects are shown in Table 2. The consumption of the protein drinks was well accepted by most of the participants and palatability ratings (evaluation scale from best to worst, 1.0 to 6.0) differed slightly between MPI (2.2) and LPI drinks (2.7).

**Table 2:** Baseline characteristics of the 33 hypercholesterolemic subjects participating in both 8 wk intervention periods

**Randomization groups**	**Group AB**	**Group BA**	***P***^**a**^
n	16	17	–
Age (y)	49.7 ± 12.8	49.4 ± 13.9	0.91
Females (n)	6	12	–
Current smoking (%)	33.0	16.7	–
Physical activity ≥ 2 h/wk (%)	66.7	66.7	–
**Anthropometric data**			
Body height (m)	1.7 ± 0.1	1.7 ± 0.1	0.44
Body weight (kg)	84.2 ± 25.3	76.4 ± 14.4	0.30
BMI (kg/m^2^)	28.8 ± 6.5	27.3 ± 5.4	0.48
Body fat (kg)	24.3 ± 12.1	24.9 ± 9.0	0.86
**Blood pressure**			
Systolic BP (mm Hg)	143.9 ± 15.8	142.4 ± 17.3	0.81
Diastolic BP (mm Hg)	87.9 ± 12.0	85.7 ± 9.6	0.56
Pulse at rest (min^-1^)	69.6 ± 15.3	71.0 ± 13.6	0.78
**Plasma parameters**			
Total cholesterol (mmol/L)	6.14 ± 1.00	6.88 ± 1.16	0.06
LDL cholesterol (mmol/L)	4.02 ± 1.09	4.69 ± 1.13	0.10
HDL cholesterol (mmol/L)	1.33 ± 0.37	1.58 ± 0.49	0.11
Triacylglyceroles (mmol/L)	2.03 ± 1.50	1.55 ± 0.79	0.28
LDL:HDL cholesterol ratio	3.33 ± 1.43	3.30 ± 1.40	0.95
Urea (mmol/L)	5.45 ± 1.37	4.58 ± 1.13	0.05
hs-CRP^b^ (mg/L)	1.83 ± 2.30	2.02 ± 2.07	0.84

Values are presented as mean ± standard deviation, number or percentage.

Abbreviation: *Group AB* subjects receiving LPI in the first intervention period; *Group BA* subjects receiving LPI in the second intervention period; *LPI* lupin protein isolate, *BP* blood pressure, *hs*-*CRP* high-sensitivity C-reactive protein.

^a^*P*-value is for differences between randomization groups using independent samples *t*-test.

^b^hs-CRP values of some participants had to be excluded due to a temporary inflammatory status at time of measurement; group AB; n = 10; group BA, n = 13.

### Nutrient intake

The analysis of the 5 d food record provided information regarding the composition of the diet at baseline. Energy and carbohydrate intakes were in accordance with reference values, whereas protein, fat, and cholesterol intakes were higher compared to recommended values [19] (Table 3). Nutrient intake following consumption of the 2 d standard diet at wk 8 did not differ between the two treatments LPI and MPI. In comparison to the diet at baseline, the intake of energy, protein, and fat was significantly raised during the standard diet for both protein interventions (*P* ≤ 0.028).

**Table 3:** Nutrient intake calculated from the 5 d food record at baseline and from the 2 d standard diet after 8 wk of intervention with LPI and MPI

				**LPI**	**MPI**	
			***Food record***	***Standard diet***	***Standard diet***	
				**Changes from baseline**	**Changes from baseline**	***P***^**a**^
**Nutrient**	**D-A-CH**^**b**^		**Baseline**	**wk 8**	**wk 8**	**wk 8**
Energy (MJ/d)	(m) 10.5	(f) 8.5	9.8 ± 2.6	1.21 ± 2.18^*^	1.3 ± 2.1^*^	0.78
Protein (g/d)	(m) 58.0	(f) 46.0	84.4 ± 25.9	22.8 ± 20.6^***^	24.6 ± 20.7^***^	0.69
Fat (g/d)	(m) 77.0	(f) 60.0	94.3 ± 34.5	18.5 ± 29.6^**^	19.7 ± 28.6^**^	0.80
Carbohydrates (g/d)	(m) 288	(f) 225	259 ± 78	16 ± 68	18 ± 71	0.83
Cholesterol (mg/d)	(m) < 300	(f) < 300	374 ± 170	−57 ± 140	−49 ± 137	0.69

Values are presented as mean ± standard deviation, n = 33.

Abbreviation: *LPI* lupin protein isolate, *MPI* milk protein isolate, *m* males, *f* females.

^a^*P*-value is for differences between treatments at wk 8 determined by independent samples *t*-test.

^b^Reference values from D-A-CH (2004) for males and females between 51 and 65 years.

^*, **, ***^Significant differences comparing wk 8 with baseline determined by independent samples *t*-test (^*^P ≤ 0.050, ^**^P ≤ 0.010, ^***^P ≤ 0.001).

### Anthropometric data and blood pressure

No treatment effects in anthropometric data or blood pressure were seen at wk 8 among the two protein interventions (Table 4). Compared to baseline, there was a slight increase in body weight and body fat after 8 wk of intervention with both LPI and MPI (*P* ≤ 0.045). Systolic blood pressure was significantly reduced after 8 wk of the two protein interventions (*P* ≤ 0.014). Diastolic blood pressure and pulse at rest were decreased after 8 wk of LPI intervention (*P* ≤ 0.044), whereas they remained constant throughout the MPI intervention.

**Table 4:** Anthropometric data at baseline and changes after 8 wk of intervention with LPI and MPI

		**LPI**	**MPI**	
		**Changes from baseline**	**Changes from baseline**	***P***^**a**^
	**Baseline**	**wk 8**	**wk 8**	**wk 8**
Body weight (kg)	80.0 ± 20.2	0.6 ± 1.6^*^	0.7 ± 1.5^**^	0.61
BMI (kg/m^2^)	28.0 ± 5.9	0.2 ± 0.6^*^	0.2 ± 0.5^**^	0.67
Body fat (kg)	24.6 ± 10.3	0.5 ± 1.3^*^	0.6 ± 1.2^**^	0.65
Systolic BP (mm Hg)	143.1 ± 16.4	−8.4 ± 13.6^***^	−5.9 ± 12.9^*^	0.29
Diastolic BP (mm Hg)	86.7 ± 10.7	−2.7 ± 7.5^*^	−1.5 ± 7.7	0.31
Pulse at rest^b^ (min^-1^)	70.4 ± 14.2	−4.0 ± 10.8^*^	−1.8 ± 7.6	0.16

Values are presented as mean ± standard deviation, n = 33.

Abbreviation: *LPI* lupin protein isolate, *MPI* milk protein isolate, *BP* blood pressure.

^a^*P*-value is for differences between treatments at wk 8 determined by repeated measures ANOVA.

^b^Data were not normally distributed and/or had heterogeneous variances, and thus were statistically analyzed using a linear mixed model.

^*, **, ***^ Significant differences comparing wk 8 with baseline determined by repeated measures ANOVA (^*^P ≤ 0.050, ^**^P ≤ 0.010, ^***^P ≤ 0.001).

### Plasma parameters

Plasma lipid parameters did not differ between the two treatments, neither at wk 4 nor wk 8, except for a higher HDL cholesterol concentration at wk 4 following intervention with LPI (*P* = 0.036, Table 5). Compared to baseline, after 4 wk but not after 8 wk, there was a decrease in LDL cholesterol following both protein interventions (*P* ≤ 0.008) and in LDL:HDL cholesterol ratio following LPI intervention (*P* = 0.003). Concentrations of total cholesterol and triacylglyceroles were not significantly affected by the protein interventions, except for an increase in triacylglyceroles after 8 wk of LPI intervention (*P* = 0.022).

**Table 5:** Plasma concentrations of blood lipids, hs-CRP, and urea at baseline and changes after 4 wk and 8 wk of intervention with LPI and MPI

		**LPI**		**MPI**			
		**Changes from baseline**		**Changes from baseline**		***P***^***a***^	***P***^***a***^
	**Baseline**	**wk 4**	**wk 8**	**wk 4**	**wk 8**	**wk 4**	**wk 8**
Total cholesterol (mmol/L)	6.54 ± 1.14	−0.12 ± 0.48	−0.05 ± 0.44	−0.22 ± 0.63	0.02 ± 0.49	0.36	0.52
LDL cholesterol (mmol/L)	4.38 ± 1.14	−0.26 ± 0.53^**^	−0.08 ± 0.50	−0.32 ± 0.54^**^	−0.06 ± 0.34	0.47	0.90
HDL cholesterol (mmol/L)	1.46 ± 0.45	0.04 ± 0.15	−0.05 ± 0.19	−0.03 ± 0.18	−0.02 ± 0.13	0.036	0.20
Triacylglyceroles (mmol/L)	1.77 ± 1.17	0.08 ± 0.59	0.19 ± 0.45^*^	0.17 ± 0.70	0.16 ± 0.77	0.59	0.77
LDL:HDL cholesterol ratio	3.32 ± 1.39	−0.29 ± 0.53^**^	0.02 ± 0.53	−0.20 ± 0.62	−0.05 ± 0.39	0.34	0.29
hs-CRP^b, c^ (mg/L)	1.93 ± 2.12	−0.23 ± 1.29	−0.28 ± 1.38	−0.09 ± 1.17	−0.32 ± 1.41	0.63	0.89
Urea (mmol/L)	4.98 ± 1.30	0.59 ± 0.94^***^	0.47 ± 1.10^*^	0.78 ± 1.19^***^	0.44 ± 1.06^*^	0.36	0.86

Values are presented as means ± standard deviation, n = 33.

Abbreviation: *LPI* lupin protein isolate, *MPI* milk protein isolate, *hs*-*CRP* high-sensitivity C-reactive protein.

^a^*P*-value is for differences between treatments at wk 4 or at wk 8 determined by repeated measures ANOVA.

^b^Data were not normally distributed and/or had heterogeneous variances, and thus were statistically analyzed using a linear mixed model.

^c^ n = 23, hs-CRP values of some participants had to be excluded due to a temporary inflammatory status at time of measurement.

^*, **, ***^Significant differences comparing wk 4 and wk 8 with baseline determined by repeated measures ANOVA (^*^P ≤ 0.050, ^**^P ≤ 0.010, ^***^P ≤ 0.001).

**Figure 2:**
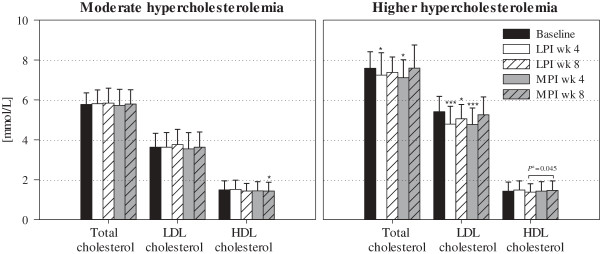
**Plasma cholesterol concentrations [mmol/L] at baseline and after 4 wk and 8 wk of intervention with LPI and MPI in subjects with moderate or higher hypercholesterolemia.** The study population was differentiated into two subgroups based on the total cholesterol concentration at baseline. Subjects with a cholesterol concentration ≤ 6.6 mmol/L were considered to have moderate hypercholesterolemia (n = 19); subjects with a cholesterol concentration > 6.6 mmol/L were considered to have higher hypercholesterolemia (n = 14). LPI, lupin protein isolate; MPI, milk protein isolate. ^a^*P*-value is for difference between treatments at wk 8 determined by repeated measures ANOVA. ^*, ***^ Significant differences comparing wk 4 and wk 8 with baseline determined by repeated measures ANOVA (^*^P ≤ 0.050, ^***^P ≤ 0.001).

Considering the total cholesterol concentrations at baseline, subjects with a higher initial total cholesterol (> 6.6 mmol/L, n = 14) at an average of 7.6 mmol/L showed a significant decrease in total and LDL cholesterol after 4 wk of both interventions compared to baseline (LPI: –0.34 ± 0.59 mmol/L and –0.62 ± 0.52 mmol/L; MPI: –0.47 ± 0.76 mmol/L and –0.64 ± 0.55 mmol/L; *P* ≤ 0.048; Figure 2). After 8 wk, a reduction of LDL cholesterol, but not of total cholesterol, was present following LPI intervention (–0.35 ± 0.54 mmol/L; *P* = 0.032). LDL:HDL cholesterol ratios were significantly decreased after 4 wk of LPI and MPI intervention (LPI: –0.68 ± 0.52 mmol/L; MPI: –0.52 ± 0.64 mmol/L; *P* ≤ 0.009; data not shown). In contrast, in subjects with moderately elevated initial total cholesterol (≤ 6.6 mmol/L, n = 19) at an average of 5.8 mmol/L, no changes in total and LDL cholesterol (Figure 2) as well as in LDL:HDL cholesterol ratio (data not shown) could be observed, both after 4 wk and after either 8 wk of LPI or MPI intervention.

There were no significant differences between the two treatments with respect to hs-CRP and urea in plasma at wk 4 or wk 8 (Table 5). Concentrations of hs-CRP decreased after 4 wk and 8 wk of LPI as well as MPI intervention compared to baseline. However, these differences did not reach statistical significance (*P* ≤ 0.82). Compared to baseline, the concentrations of plasma urea were increased after 4 wk of both protein interventions (*P* ≤ 0.001). Following 8 wk of both protein interventions, plasma urea was still higher compared to baseline (*P* ≤ 0.022), however, the magnitude was smaller than after 4 wk of intervention.

### Serum amino acids

Serum amino acid concentrations at baseline, treatment effects, and changes over time are shown in Table 6. Except for aspartate, glycine and arginine, the concentrations of proteinogenic amino acids were significantly higher at wk 4 (*P* ≤ 0.042) for MPI relative to LPI. However, there were no significant differences between the two treatments at wk 8. Compared to baseline, the concentrations of almost all single amino acids increased after 4 wk, but not after 8 wk of intervention with MPI (*P* ≤ 0.019). Following the 4 wk and 8 wk intervention with LPI, most of the amino acid concentrations remained unchanged.

**Table 6:** Serum concentrations of amino acids at baseline and changes after 4 wk and 8 wk of intervention with LPI and MPI

		**LPI**		**MPI**			
		**Changes from baseline**		**Changes from baseline**		***P***^***a***^	***P***^***a***^
**Amino acid (μmol/L)**	**Baseline**	**wk 4**	**wk 8**	**wk 4**	**wk 8**	**wk 4**	**wk 8**
Alanine	425.0 ± 147.6	−1.1 ± 98.2	1.8 ± 132.2	45.1 ± 102.0^*^	2.6 ± 121.8	0.022	0.96
Arginine	96.2 ± 30.3	1.0 ± 24.7	9.0 ± 28.4	4.6 ± 28.3	3.1 ± 29.8	0.43	0.18
Asparagine	48.9 ± 13.9	2.8 ± 16.0	7.2 ± 16.3^*^	7.8 ± 12.5^***^	3.1 ± 12.1	0.08	0.09
Aspartate^b^	19.6 ± 8.9	−4.0 ± 10.5^*^	−6.2 ± 7.5^***^	−1.1 ± 8.5	−6.8 ± 8.9^***^	0.22	0.56
Cystine	65.2 ± 18.5	−3.3 ± 12.5	1.9 ± 16.0	2.2 ± 12.4	1.4 ± 15.9	0.025	0.81
Glutamine	623.7 ± 169.6	−22.7 ± 105.8	14.2 ± 132.0	30.6 ± 132.1	19.0 ± 150.4	0.026	0.84
Glutamate^b^	55.5 ± 27.8	−9.3 ± 25.2^*^	−14.6 ± 20.3^**^	3.3 ± 28.9	−15.1 ± 22.0^**^	0.038	0.89
Glycine	246.1 ± 82.4	−14.5 ± 50.5	−10.6 ± 63.8	0.8 ± 58.1	−13.4 ± 66.8	0.17	0.76
Histidine	90.5 ± 26.1	−4.9 ± 19.0	6.9 ± 21.5	8.2 ± 21.6	3.5 ± 23.3	0.001	0.24
Isoleucine	63.5 ± 32.0	3.2 ± 28.2	4.2 ± 27.6	13.4 ± 31.3^*^	2.9 ± 28.3	0.014	0.70
Leucine	145.2 ± 48.4	0.9 ± 40.9	7.0 ± 39.6	24.5 ± 48.1^**^	6.4 ± 41.8	0.002	0.91
Lysine	200.2 ± 57.2	−8.4 ± 50.7	9.8 ± 53.3	29.7 ± 58.0^**^	18.0 ± 58.4	0.0001	0.29
Methionine	22.9 ± 7.8	−1.9 ± 7.1	0.4 ± 7.4	3.8 ± 8.3^*^	0.5 ± 7.8	< 0.0001	0.96
Phenylalanine	66.8 ± 16.4	−1.4 ± 15.8	1.7 ± 18.5	8.4 ± 18.0^*^	1.6 ± 18.4	0.003	0.98
Proline^b^	169.7 ± 99.9	18.8 ± 112.9	24.5 ± 101.0	81.8 ± 184.1^**^	39.7 ± 117.2	0.007	0.31
Serine^b^	112.4 ± 35.6	−9.8 ± 29.2	−3.8 ± 30.8	5.1 ± 28.9	−4.6 ± 27.0	0.042	0.87
Threonine^b^	132.5 ± 49.7	−6.0 ± 35.8	2.2 ± 39.7	14.6 ± 37.1	7.7 ± 40.8	0.013	0.38
Tryptophan	76.0 ± 27.7	−7.3 ± 25.2	9.4 ± 44.9	4.8 ± 28.6	−1.3 ± 28.2	0.010	0.13
Tyrosine	75.2 ± 22.8	−0.5 ± 18.8	4.2 ± 21.3	16.0 ± 27.7^**^	4.3 ± 19.0	0.002	0.98
Valine	243.5 ± 87.3	−3.8 ± 75.8	13.4 ± 64.5	45.6 ± 78.1^**^	24.6 ± 79.2	0.0004	0.29
EAA	1041.1 ± 313.0	−29.5 ± 261.3	54.9 ± 254.4	153.2 ± 302.9^**^	63.8 ± 297.5	0.0003	0.79
Non-EAA	1937.4 ± 507.4	−42.3 ± 382.2	27.7 ± 458.8	196.2 ± 478.1^*^	33.2 ± 458.2	0.005	0.93
BCAA	452.2 ± 165.4	0.3 ± 142.1	24.6 ± 126.8	83.6 ± 154.7^**^	33.9 ± 145.3	0.001	0.62
Lysine:arginine ratio	2.16 ± 0.55	−0.14 ± 0.32^*^	−0.04 ± 0.28	0.17 ± 0.35^**^	0.10 ± 0.34	0.0004	0.16

Values are presented as means ± standard deviation, n = 33.

Abbreviation: *LPI* lupin protein isolate, *MPI* milk protein isolate, *EAA* essential amino acids, *Non*-*EAA* non-essential essential amino acids, *BCAA* branched chained amino acids.

^a^*P*-value is for differences between treatments at wk 4 or at wk 8 determined by repeated measures ANOVA.

^b^ Data were not normally distributed and/or had heterogeneous variances, and thus were statistically analyzed using a linear mixed model.

^*, **, ***^ Significant differences comparing wk 4 and wk 8 with baseline determined by repeated measures ANOVA (^*^P ≤ 0.050, ^**^P ≤ 0.010, ^***^P ≤ 0.001).

## Discussion

This randomized crossover study reveals that a modest amount comprising 25.0 g/d of additionally consumed LPI is capable of lowering total (−5%) and LDL cholesterol concentrations (–12%) as well as the LDL:HDL cholesterol ratio (−16%) from baseline to wk 4, primarily in subjects with higher hypercholesterolemia (> 6.6 mmol/L).

The lipid-lowering activity of dietary treatments appears to be strongly dependent on the subjects’ initial cholesterol concentrations [20,21]. A meta-analysis of 38 human studies on soy protein ascertained that the net changes in total as well as in LDL cholesterol after intervention were directly related to the total cholesterol concentration at baseline [20]. A more recent re-evaluation by Sirtori *et al*. [21] which included a further 33 studies on soy protein confirmed this dependency. In line with this, a subgroup analysis within the present study revealed that the total and LDL cholesterol-lowering activities of LPI and MPI were restricted to subjects with higher initial total cholesterol with an average of 7.6 mmol/L (Figure 2) indicating that there is a similar dependency between cholesterol-lowering activity and baseline cholesterol concentrations for lupin protein.

As reviewed by Anderson and Konz [22], a 1% increase in either total or LDL cholesterol increases the risk for coronary heart disease by 2% to 3% and 1%, respectively. Thus, after a 4 wk LPI intervention the risk for cardiovascular events such as coronary heart diseases would be reduced by 10% to 15% in subjects with higher hypercholesterolemia. Due to the lack of change in subjects with moderate hypercholesterolemia, the lipid-lowering effects for the whole study population were lower. The overall changes in plasma cholesterol after 4 wk of intervention are consistent with two other studies that show a LDL cholesterol- [8] and a moderate total cholesterol-lowering activity [8,9] of 35.0 g/d lupin protein consumed by hypercholesterolemic subjects over a short-term period of 4 wk or 6 wk. A recent study conducted by our workgroup in hypercholesterolemic subjects revealed a decrease in the LDL:HDL cholesterol ratio by 7% after consumption of 40.0 g/d LPI over 8 wk, whereas total and LDL cholesterol were not altered [12]. In contrast, Belski *et al*. [23] and Hodgson *et al*. [24] did not find changes in plasma lipid concentrations in overweight or obese participants following a long-term intervention from 16 wk to twelve months with an *ad libitum* diet higher in protein and fiber obtained by enriching foods with lupin flour.

The present study showed a significant reduction in systolic blood pressure by 8.4 mm Hg after 8 wk of LPI intervention. Since an increase of 1 mm Hg in systolic blood pressure is expected to increase the risk for coronary heart disease by 2.4% [22] the observed effect in our study could reduce the risk by 20%. These results are consistent with three previous studies that found a significant decline in blood pressure following consumption of lupin protein [7] or lupin flour [23,25].

Similar to the findings of Weisse *et al*. [8], LPI intervention *per se* only minimally changed amino acid profile (Table 6). The concentrations of methionine were decreased by 8% after 4 wk equivalent to the observed 7% decline in the study by Weisse *et al*. [8]. Since LPI relative to MPI had almost a threefold higher arginine and half the lysine proportion (Table 1), at wk 4, but not at wk 8 of LPI intervention, the lysine:arginine ratio in serum was significantly lower compared to MPI (Table 6).

Evidently, there was a general decrease in the extent of physiological effects of both protein interventions from wk 4 to wk 8. This aspect may be explained by a declining compliance to the study protocol after 4 wk of intervention, which is supported by a decrease in plasma urea from wk 4 to wk 8 (Table 5). As there was an increase in energy intake (Table 3) as well as in body weight and body fat (Table 4) after 8 wk of both protein interventions compared to baseline, we can presume that the majority of subjects did not replace an *iso*-caloric part of their usual diet with the protein drinks. A decrease in body weight is associated with lower concentrations of triacylglyceroles, total and LDL cholesterol as well as with higher HDL cholesterol [26]. Thus, the observed weight gain might have additionally contributed to a worsening of the lipid profile from wk 4 to wk 8.

There were no significant differences in the plasma concentrations of cholesterol and of hs-CRP or in blood pressure between LPI and MPI intervention. This lack of treatment effects is not entirely surprising since several studies attribute milk proteins, particularly several milk peptides, with beneficial physiological properties such as hypocholesterolemic, hypotensive, and anti-inflammatory activities [3]. Furthermore, increasing evidence indicates that the substitution of protein from animal as well as plant sources at the expense of carbohydrates may beneficially affect plasma lipids [4], facilitates loss of body weight [27] and body fat [28], and can lower blood pressure [5]. The mechanisms and bioactive components of lupin protein responsible for the beneficial effects in the human body have not yet been elucidated [6]. Contrary to soy, proteins from lupin are almost free from isoflavones [29] and thus physiological effects can be attributed to the protein and/or its components *per se*. According to Rahman *et al*. [30], the low lysine:arginine ratio might be responsible for the hypocholesterolemic properties of lupin protein. As reported by Rajamohan and Kurup [31], a decrease in serum cholesterol in rats was caused by a globulin fraction of sesame seeds with a low lysine:arginine ratio of 0.67 comparable to the value determined for the LPI (0.38) used in the present study. However, studies on the impact of different lysine:arginine ratios on lipid metabolism are lacking. Notably, the high proportion of arginine amounting to around 10% in lupin protein should be taken into consideration with regard to the physiological impact. Recent studies indicate that arginine is capable of modulating the concentrations of lipid signaling molecules [32,33] and the expression of genes involved in the regulation of lipid homeostasis [32] which might lead to changes in the concentration of cholesterol. Hurson *et al*. [34] investigated the effect of an oral supplementation with 17 g arginine over 2 wk in elderly subjects. In the arginine-supplemented group, total cholesterol significantly decreased by 10% due mainly to reduced LDL cholesterol (-10%), whilst HDL cholesterol remained constant. These observed changes in cholesterol concentrations are in accordance with the results of the present study after 4 wk of 25 g/d LPI intervention. However, in the current study, the arginine uptake of 2.5 g/d *via* LPI was much lower than the supplemented 17 g/d arginine in the study by Hurson *et al*. [34]. Lupin protein seems to affect the expression of hepatic genes involved in lipid metabolism as previously shown in hypercholesterolemic rats [35,36] and, further, to alter the activity of LDL receptor as shown in a human hepatoma cell line [29]. Supporting these results, Weisse *et al*. [8] observed an increase in mRNA abundance of the sterol regulatory element-binding protein-2 and LDL receptor along with a decrease in mRNA concentrations of 3-hydroxy-3-methylglutaryl-CoA reductase in mononuclear blood cells from hypercholesterolemic subjects after 6 wk intervention with 35 g/d lupin protein. Apart from the specific amino acid profile of lupin protein, several bioactive peptides as well as entire proteins are equally capable of demonstrating favorable properties [6].

In the present study, we could not detect a triacylglycerole-lowering activity of LPI. Thus, the inconsistent experimental data referring to the effect of lupin protein on triacylglyceroles [7-9] indicates the necessity of an inclusion of this parameter in further human studies. Furthermore, in future studies, it may be desirable to incorporate the test proteins in usual dietary foods in order to increase the subjects’ compliance and to avoid changes in dietary composition, thereby sustain body weight and body composition over the whole study time.

## Conclusion

The present study suggests that the supplementation of 25 g/d LPI positively affects LDL cholesterol and LDL:HDL cholesterol ratio particularly in subjects with higher hypercholesterolemia. These beneficial effects, were however, largely absent after 8 wk of intervention, due most likely to a declining compliance from wk 4 to wk 8.

Based on our results, we do not expect any adverse effects of lupin protein when integrated in human nutrition above all because the protein is almost free from isoflavones. Lupin therefore can be considered as an alternative and valuable source of plant protein with respect to soy protein. Moreover, supplementation of modest amounts of lupin protein into the diet could provide a safe and non-pharmacological approach of attenuating the extent of hypercholesterolemia, thereby reducing the risk of cardiovascular diseases.

## Abbreviations

LPI: Lupin protein islolate; MPI: Milk protein islolate; hs-CRP: High-sensitivity C-reactive protein.

## Competing interests

The authors declare that they have no personal or financial conflict of interests.

## Authors’ contributions

MB, AF and GJ designed the research; MB was responsible for supervising the study, sample handling, coordination and conduction of the analyses; MB, JK, and MK analyzed data; MB performed statistical analysis; MB and GJ were responsible for data interpretation and had primary responsibility for final content; MB wrote the paper; all authors read and approved the final manuscript.
